# Associations of dietary patterns and screen time with depressive symptoms among adolescents in Shandong Province, China

**DOI:** 10.1186/s12889-025-25976-z

**Published:** 2025-12-20

**Authors:** Xiaomei Jiang, Zhongyou Li, Pingjing Wen, Yiyi Ling, Hai Li, Jiongli Huang

**Affiliations:** Department of Preventive Medicine, School of Public Health and Management, Guangxi University of Chinese Medicine, Nanning, 530200 China

**Keywords:** Adolescents, Depressive symptoms, Dietary patterns, Screen time, Combined exposure

## Abstract

**Background:**

Understanding the interplay among dietary patterns, screen time, and depressive symptoms among adolescents is crucial for the development of effective prevention strategies. This study aimed to explore the associations between dietary patterns, screen time, and their combined effects on depressive symptoms among high school students in Shandong Province, China.

**Methods:**

Utilizing the Database of Youth Health (DYH) from the China Population Health Science Data Center in 2020, demographic characteristics, food intake frequencies, and screen time information were collected from 8,276 high school students aged 12–20 years in Shandong Province, China. Principal component analysis was employed to identify distinct dietary patterns, and logistic regression analysis was used to assess the associations of dietary patterns and screen time single and co-exposure with depressive symptoms.

**Results:**

The prevalence of depressive symptoms among adolescents in Shandong Province stood at 18.9%. Principal component analysis identified three main dietary patterns: balanced dietary patterns, high-protein dietary patterns, and processed dietary patterns. In comparison to students who maintained balanced dietary patterns, those who followed processed dietary patterns demonstrated a higher risk of depressive symptoms, exhibiting odds ratios (ORs) and 95% confidence intervals (CIs) of 1.62 (1.40–1.88). Additionally, adolescents whose screen time ≥ 2 h/d faced a greater risk of depressive symptoms than those whose screen time was < 2 h/d (OR = 1.46, 95% CI 1.25–1.70). Adolescents in high-protein dietary patterns with screen time ≥ 2 h/d, processed dietary patterns with screen time < 2 h/d, and processed dietary patterns with screen time ≥ 2 h/d all showed elevated risks of depressive symptoms. The respective ORs (95% CIs) for these groups were 1.71 (1.27–2.31), 1.60 (1.36–1.89), and 2.34 (1.86–2.94).

**Conclusions:**

Our findings demonstrate that co-exposure to high-protein dietary patterns and screen time ≥ 2 h/d is significantly associated with increased risk of depressive symptoms in adolescents. Notably, processed dietary patterns exhibit a more pronounced association, demonstrating heightened depressive symptom risks even when combined with either shorter (< 2 h/d) or longer (≥ 2 h/d) screen time durations.

**Supplementary Information:**

The online version contains supplementary material available at 10.1186/s12889-025-25976-z.

## Introduction

Depression has emerged as a severe global public health concern. It not only constitutes one of the primary contributors to the global burden of disease but also represents the leading cause of death among mental health issues [1]. According to the 2021 WHO report, approximately 280 million people worldwide suffer from depression, with approximately 700,000 deaths annually attributed to this condition [2]. Projections indicate that by 2030, depression will escalate to become the foremost global burden of disease [3]. Research showed that the global prevalence of depressive symptoms in adolescents increased from 24% between 2001 and 2010 to 37% between 2011 and 2020 [4]. Furthermore, research indicates the incidence of depression among teenagers rises rapidly after entering adolescence [5]. In China, the high incidence of adolescent depression has become increasingly prominent. The China National Mental Health Development Report [6] revealed that the number of depression patients in China exceeds 95 million, with a detection rate of 24.6% among adolescents, increasing to 40% among high school students. Some studies indicate that adolescents with depression are prone to experiencing diminished academic performance, smoking, alcohol abuse, and even suicide, which can severely negatively impact their physical and mental health [7, 8]. Depressive symptoms usually refer to a subclinical form of depression, which is defined by measuring self-reported depressive syndromes through specific boundaries [9]. In addition, research shows that depressive symptoms are a psychological disorder that has not yet reached the clinical criteria for depression, and adolescent depressive symptoms constitute an early-onset subtype of adult depression [10]. Therefore, early identification and control of risk factors for adolescent depressive symptoms are of paramount importance in the prevention and management of depression.

Adolescence is a crucial shaping period for an individual’s growth and development, as well as a critical period for the development of the brain, cognition and emotions, thus becoming a high-risk period for the development of psychological problems such as depression and anxiety among teenagers [11]. Risk factors for adolescent depression include genetics, the environment, personality traits, behaviors, etc [12–14]. With societal progress and technological advancements, the association between changes in adolescents’ lifestyle behaviors and depression has garnered considerable attention. Recent studies have demonstrated independent associations between diet and screen time and adolescent depression [15]. On the one hand, research has shown links between specific nutrients or food groups and depressive symptoms in adolescents [16]. For instance, a deficiency of vitamin D among middle school students is positively correlated with the risk of depressive symptoms [17].

Unhealthy eating was significantly associated with prevalence of anxiety, depression, and stress. Excessive intake of sweets (such as candies) and low of dairy products (milk and its derivatives) were associated with a higher prevalence of psychological [18]. A study conducted in South Korea [19] reported that excessive energy drink consumption was related to depressive moods in students aged 12 ~ 18 years. However, people’s daily diets do not consist of single nutrients or food groups but rather combinations of various foods, which may interact with each other. Dietary patterns consider the relationships among multiple nutrients, reflecting the complexity and integrity of dietary structures [20]. Consequently, in recent years, nutritional epidemiologists have emphasized the use of dietary patterns to investigate the relationship between diet and disease [21]. Although research on the associations between dietary patterns and depressive symptoms is increasing, this research direction remains relatively limited. Oddy et al. [22] reported that western dietary patterns were associated with an increased risk of mental health disorders (including depressive symptoms) in adolescents. A study conducted in China [23] revealed that modern and snack-aquatic dietary patterns were correlated with a greater risk of adolescent depressive symptoms. A recent study [24] revealed that high consumption of vegetables and poultry meat, coupled with low consumption of processed foods, was associated with a lower risk of depressive symptoms in junior high school students, while low consumption of milk, dairy products, and eggs, along with high consumption of processed foods, was associated with an increased risk of depressive symptoms in this population.

On the other hand, studies [25, 26] have demonstrated an association between screen time and depressive symptoms. With the advent of the internet era and the widespread use of electronic devices, the issue of excessive screen time among adolescents has become increasingly prominent [27]. Adolescents may become addicted to screen-based activities, such as watching videos and television, using smartphones and tablets, and playing online games [28, 29]. Prolonged screen time not only affects adolescents’ physical health, leading to myopia, obesity, insulin resistance, and sleep disturbances, but also may contribute to the onset of psychological issues, including depressive symptoms [30–35]. Most existing research has focused on the independent effects of dietary patterns or screen time on adolescent depression. However, adolescents often engage in both behaviors simultaneously. Studies examining joint exposure to dietary patterns and screen time in relation to depression are very rare. For example, Yang et al. [36] reported that junior high school students with screen time ≥ 2 h/d and unhealthy dietary behaviors had a greater risk of depressive symptoms than the sum of the individual effects of these two factors. In China, high school students face significant academic pressure due to the college entrance examination (GaoKao), which coincides with a critical stage of their physical and mental development. The prevalence of depression among this population has garnered considerable attention [37]. Shandong Province, as the second most populous province in China and a major educational hub, experiences intense competition for the gaokao, with many students participating. This results in relatively scarce educational resources and further increases students’ academic burden. The detection rate of depression among adolescents in Shandong Province has reached 45.1% [38].

In summary, most researches have been conducted on the associations between dietary patterns or screen time alone and depressive symptoms, whereas studies on the combined effects of these two factors on adolescent depressive symptoms are relatively rare. A previous study has shown that depression has a negative impact on the sleep quality, psychosocial function and academic performance of adolescents, and is regarded as an important predictor of suicidal behavior [39]. Moreover, a report indicated that high school students, who are a major group of adolescents, are at high risk of depression [6]. Based on these arguments, we explored the associations between dietary patterns and screen time (both alone and in combination) with depressive symptoms in a large sample of Chinese high school students, thereby providing effective intervention strategies for adolescent depression and promoting the development of mental health among Chinese adolescents.

## Methods

### Study population

The data originated from the Database of Youth Health (DYH) [40], the inaugural open-access dataset on Chinese adolescents’ health and related behaviors. DYH employs a multistage stratified cluster random sampling approach to ensure a representative sample. This study utilized 2020 cross-sectional survey results from DYH, examining 9404 high school students across 10 cities in Shandong Province, China (Jinan, Dongying, Weifang, Jining, Weihai, Dezhou, Liaocheng, Linyi, Heze, and Laiwu). The survey covered personal details, family circumstances, nutrition, diet, risk behaviors, mental health, and other aspects. Following data preprocessing, which included matching, handling missing values, outlier removal, and recoding, 8,276 students were included in the analysis (Fig. S1). The study was conducted in accordance with the Declaration of Helsinki and was approved by the Ethics Committee of Shandong University with issue number 20180517. All the subjects were fully informed and signed informed consent before the investigation.

### Depressive symptom assessment

The depressive symptoms of high school students were evaluated via items related to depression on the Symptom Checklist (SCL-90). The scale was developed by American psychologists Derogate [41] and is currently used in China in a revised Chinese version by Wang [42]. The SCL-90 is a practical, simple and valuable scale for mental health status identification and group mental health surveys, with Cronbach’s alpha coefficients ranging from 0.77 to 0.99 [41]. It can be used to assess mental health over a specific period of time, usually a week [42]. Five grades were used, 0 = never, 1 = mild, 2 = moderate, 3 = severe, and 4 = severe, and the scores were 1, 2, 3, 4, and 5, respectively. Depressive symptoms include 13 items, and each answer score calculates the average score of depressive symptom factors. In accordance with the recommendations of the Manual of Psychiatric Rating Scale of Modern Psychiatry Series [43], the present study determined the presence of depressive symptoms with an average score of depressive symptom factor > 2.

### Dietary patterns

A food frequency questionnaire was used to collect participants’ food intake frequency over the past week. The contents included green vegetables, red-orange vegetables (carrots, tomatoes, etc.), other vegetables, potatoes (excluding french fries, fried potatoes or potato chips), soybean products, tubers, common meat, processed meat, seafood, fruits, eggs, dairy, instant noodles, western fast food, sugar-sweetened beverages, sweetened salt and sugar snacks and desserts, and fried food, for a total of 17 items (Table S1). Based on the frequency of food consumption within the week prior to the survey, it was classified into five grades, namely “0 times/week”, “1–2 times/week”, “approximately once every other day/week”, “approximately once a day/week”, and “more than once a day/week”. Principal component analysis [16, 44] was applied to the orthogonal rotation of 17 food groups to obtain the dietary patterns of the study population.

### Screen time

Screen time refers to the average time spent on watching TV, playing video games (including mobile phones, tablets and computers), and chatting on social software such as QQ, WeChat and Facebook every day. In accordance with the recommendations of the American Association of Pediatrics [45], the daily screen time of children and adolescents should not exceed 2 h. The screen time should be divided into < 2 h/d and ≥ 2 h/d, and screen time ≥ 2 h/d should be defined as excessive screen time.

### Confounding factors

Confounding factors, including sociodemographic data (sex, age, nation, household registration type, residential location, only-child status, household economic status, paternal education level and maternal education level), risky behavior (smoking status, drinking status), physical activity status, and other status (household computer and internet access status, parental academic expectation, on-campus accommodation status, and number of close friends) were extracted from the 2020 DYH. Directed acyclic graph of the relationship between dietary patterns, screen time and depressive symptoms was shown in Fig. S2.

### Statistical analysis

SPSS software (version 26.0. IBM Inc., Armonk, NY) was used for the statistical analysis. Normal continuous variables are represented by mean ± standard deviation (x ± s), and categorical variables are represented by [n (%)]. The Chi-square test assessed associations among categorical variables. The group with balanced dietary patterns and screen time < 2 h/d was used as the reference. To explore the risk of depressive symptoms among high school students, balanced dietary patterns with screen time ≥ 2 h/d, high-protein dietary patterns with screen time < 2 h/d, high-protein dietary patterns with screen time ≥ 2 h/d, processed dietary patterns with screen time < 2 h/d, and processed dietary patterns with screen time ≥ 2 h/d were used. All tests were bilateral, and the significance level was *P*-value < 0.05.

## Results

### Principal component analysis of dietary patterns

Seventeen food groups were included in the exploratory factor analysis. Food items with factor loadings > 0.25 were considered to have meaningful contributions to the dietary patterns and were retained for interpretation. In the Bartlett sphericity test, Kaiser‒Meyer‒Olkin (KMO) = 0.921, *P* < 0.001, indicating that the variables are correlated and can be analyzed via principal component analysis (PCA) (Table 1 and Fig. S3). The cumulative contribution rate was 59.322%. In factor 1, the factor loadings of sugar-sweetened beverages, western fast food, sweetened salt and sugar snacks and desserts, fried food, instant noodles, processed meat and seafood were greater (factor loading > 0.25), and the factor loadings of sugar-sweetened beverages, western fast food, sweetened salt and sugar snacks and desserts, fried food, instant noodles, and processed meat were greater (factor loading > 0.5). This dietary pattern is similar to the western diet [46–48]; that is, it is characterized by the consumption of processed foods with high sugar, fat and energy, and it is referred to as “processed dietary patterns”. In factor 2, red-orange vegetables, green vegetables, other vegetables, tubers, potatoes, soybean products, seafood, eggs, common meat and fruits had higher factor loadings (factor loading > 0.25), especially red-orange vegetables, which had the highest load (factor loading = 0.802). The dietary patterns were dominated by plant-based foods, and animal-based foods were more common. Moreover, the intake of seafood, beans and vegetables is high, which is similar to the dietary pagoda recommended in the 2022 edition of the Dietary Guide for Chinese Residents [49], and it is named “balanced dietary patterns”. In factor 3, the factor loadings of eggs, dairy, common meat, fruits, processed meat, green vegetables, soybean products and seafood were all > 0.25; in particular, the factor loading of milk was the highest (factor loading = 0.799). The dietary patterns were characterized by high protein content. Geeta Trilok-Kumar et al. [50]. reported a similar food group. We call this “high-protein dietary patterns”.

**Table 1:** Factor model and component load table

Foods	Processed dietary patterns	Balanced dietary patterns	High-protein dietary patterns
Sugar-sweetened beverages	0.837	0.071	0.064
Western fast food	0.797	0.025	0.048
Sweetened salt and sugar snacks and desserts	0.787	−0.016	0.164
Fried food	0.787	0.030	0.092
Instant noodles	0.728	0.145	−0.086
Processed meat	0.547	0.196	0.357
Red-orange vegetable	0.034	0.802	0.200
Tubers	0.289	0.739	0.154
Potatoes	0.197	0.717	0.115
Other vegetables	0.025	0.716	0.283
Green vegetables	−0.114	0.651	0.308
Soybean products	0.242	0.649	0.318
Seafood	0.419	0.507	0.318
Dairy	0.125	0.141	0.799
Eggs	0.089	0.302	0.667
Common meat	0.156	0.325	0.637
Fruits	−0.022	0.393	0.588
Eigenvalue	3.82	3.80	2.47
% of Variance	22.49	22.34	14.51
Cumulative%	22.49	44.83	59.33

### Sample sociodemographic and lifestyle characteristics

Table 2 presents the participant characteristics of 8,276 high school students. The participants comprised 3,949 males (47.7%) and 4,327 females (52.3%), with a mean age of 16.11 ± 1.24 years. Notably, 1,562 adolescents (18.9%) screened positive for depressive symptoms. Comparative analysis revealed significant associations between depressive symptoms and the following factors: female sex, agricultural household registration, residence in urban-rural fringe areas, non-only child status, lower household economic status, lower paternal and maternal education levels, lack of computer or internet access, parental absence of academic performance requirements, limited close friendships, smoking and alcohol consumption, lower physical activity level, processed food dietary patterns, and screen time ≥ 2 h/d (all *P-*value*s* < 0.05).

**Table 2:** Participant characteristics [n (%)]

Groups	Total (*n* = 8276)	Depressive symptoms		χ ^2^	*P*-value
		Yes (*n* = 1562)	No (*n* = 6714)		
Sex				22.49	< 0.001
Male	3949 (47.7)	661 (16.7)	3288 (83.3)		
Female	4327 (52.3)	901 (20.8)	3426 (79.2)		
Age (years)				3.77	0.287
≤ 15	2215 (26.8)	393 (17.7)	1822 (82.3)		
16	2861 (34.6)	548 (19.2)	2313 (80.8)		
17	2272 (27.5)	430 (18.9)	1842 (81.1)		
≥ 18	928 (11.2)	191 (20.6)	737 (79.4)		
Nation				0.13	0.718
Ethnic Han	8121 (98.1)	1531 (18.9)	6590 (81.1)		
Others	155 (1.9)	31 (20.0)	124 (80.0)		
Household registration type				10.16	0.001
Agricultural	5258 (63.5)	1047 (19.9)	4211 (80.1)		
Non-agricultural	3018 (36.5)	515 (17.1)	2503 (82.9)		
Residential location				11.33	0.010
City and county urban areas	3854 (46.6)	680 (17.6)	3174 (82.4)		
Urban-rural fringes	624 (7.5)	142 (22.8)	482 (77.2)		
Towns and townships	1797 (21.7)	342 (19.0)	1455 (81.0)		
Rural areas	2001 (24.2)	398 (19.9)	1603 (80.1)		
Only-child status				6.94	0.008
Yes	2365 (28.6)	404 (17.1)	1961 (82.9)		
No	5911 (71.4)	1158 (19.6)	4753 (80.4)		
Household economic status				130.75	< 0.001
Low	1291 (15.6)	383 (29.7)	908 (70.3)		
Moderate	6380 (77.1)	1042 (16.3)	5338 (83.7)		
High	605 (7.3)	137 (22.6)	468 (77.4)		
Paternal education level				75.41	< 0.001
Primary and below	958 (11.6)	273 (28.5)	685 (71.5)		
Junior high school	3423 (41.4)	640 (18.7)	2783 (81.3)		
High school and technical secondary school	2404 (29.0)	427 (17.8)	1977 (82.2)		
Junior college or above	1491 (18.0)	222 (14.9)	1269 (85.1)		
Maternal education level				76.92	< 0.001
Primary and below	1853 (22.4)	469 (25.3)	1384 (74.7)		
Junior high school	3162 (38.2)	588 (18.6)	2574 (81.4)		
High school and technical secondary school	2067 (25.0)	336 (16.3)	1731 (83.7)		
Junior college or above	1194 (14.4)	169 (14.2)	1025 (85.8)		
Household computer and internet access status				63.40	< 0.001
No computer or internet	663 (8.0)	201 (30.3)	462 (69.7)		
Computer only	334 (4.0)	69 (20.7)	265 (79.3)		
Both computer and internet	7279 (88.0)	1292 (17.7)	5987 (82.3)		
Parental academic expectation				34.43	< 0.001
Top 5 in the class	1601 (19.3)	347 (21.7)	1254 (78.3)		
Above average level	4060 (49.1)	670 (16.5)	3390 (83.5)		
Average level	1062 (12.8)	200 (18.8)	862 (81.2)		
No explicit expectation	1553 (18.8)	345 (22.2)	1208 (77.8)		
On-campus accommodation status				3.02	0.082
Yes	5649 (68.3)	1095 (19.4)	4554 (80.6)		
No	2627 (31.7)	467 (17.8)	2160 (82.2)		
Number of close friends				10.02	0.007
0 ~ 1	2966 (35.8)	613 (20.7)	2353 (79.3)		
2 ~ 4	3416 (41.3)	603 (17.7)	2813 (82.3)		
≥ 5	1894 (22.9)	346 (18.3)	1548 (81.7)		
Smoking status				235.24	< 0.001
Yes	945 (11.4)	352 (37.2)	593 (62.8)		
No	7331 (88.6)	1210 (16.5)	6121 (83.5)		
Drinking status				348.45	< 0.001
Yes	1817 (22.0)	618 (34.0)	1199 (66.0)		
No	6459 (78.0)	944 (14.6)	5515 (85.4)		
Physical activity status (times/week)				192.89	< 0.001
< 1	2013 (24.3)	589 (29.3)	1424 (70.7)		
1 ~ 2	3757 (45.4)	612 (16.3)	3145 (83.7)		
3 ~ 4	1490 (18.0)	201 (13.5)	1289 (86.5)		
≥ 5	1016 (12.3)	160 (15.7)	856 (84.3)		
Dietary patterns				169.51	< 0.001
Balanced type	2655 (32.1)	375 (14.1)	2280 (85.9)		
High-protein type	2908 (35.1)	459 (15.8)	2449 (84.2)		
Processed type	2713 (32.8)	728 (26.8)	1985 (73.2)		
Screen time (h/d)				96.51	< 0.001
< 2	7083 (85.6)	1214 (17.1)	5869 (82.9)		
≥ 2	1193 (14.4)	348 (29.2)	845 (70.8)		

### Associations of dietary patterns and screen time exposure alone with depressive symptoms in adolescents

Table 3 and Table S2 presents multivariable-adjusted analyses of dietary patterns and screen time exposure in relation to depressive symptomatology. Compared to the balanced dietary patterns group, adolescents with high-protein dietary patterns showed no statistical association with depressive symptoms (*P-*value > 0.05). However, adolescents with processed dietary patterns demonstrated 62% greater odds of depressive symptoms compared to students with balanced dietary patterns (OR = 1.62; 95% CI: 1.40–1.88). Additionally, adolescents with screen time ≥ 2 h/d demonstrated 46% greater odds of depressive symptoms compared to students with screen time < 2 h/d (OR = 1.46; 95% CI: 1.25–1.70). In addition, the results indicate female, smoking, and alcohol consumption were associated with an increased risk of depressive symptoms. In contrast, only child, and higher paternal education level (high school above), parental academic expectations above average, the number of close friends from 2 to 4, and engaging in physical activity at least once per week were associated with a lower risk of depressive symptoms.

**Table 3:** Logistic regression analysis of dietary patterns, screen time and depressive symptoms among high school students

Variables	Model 1		Model 2	
	OR (95%CI)	*P*-value	OR (95%CI)	*P*-value
Dietary patterns				
Balanced dietary patterns	1.00		1.00	
High-protein dietary patterns	1.14 (0.98–1.32)	< 0.083	1.13 (0.97–1.33)	0.112
Processed dietary patterns	2.23 (1.94–2.56)	< 0.001	1.62 (1.40–1.88)	< 0.001
Screen time (h/d)				
< 2	1.00		1.00	
≥ 2	1.99 (1.73–2.29)	< 0.001	1.46 (1.25–1.70)	< 0.001

Model 1 has no adjustment variable. Model 2 is adjusted for sex, household registration type, residential location, only-child status, household economic status, paternal education level, maternal education level, household computer and internet access status, parental academic expectation, number of close friends, smoking status, drinking status, physical activity status, and screen time/dietary patterns.

### Associations between dietary patterns combined screen time exposure and depressive symptoms in adolescents

**Fig. 1:**
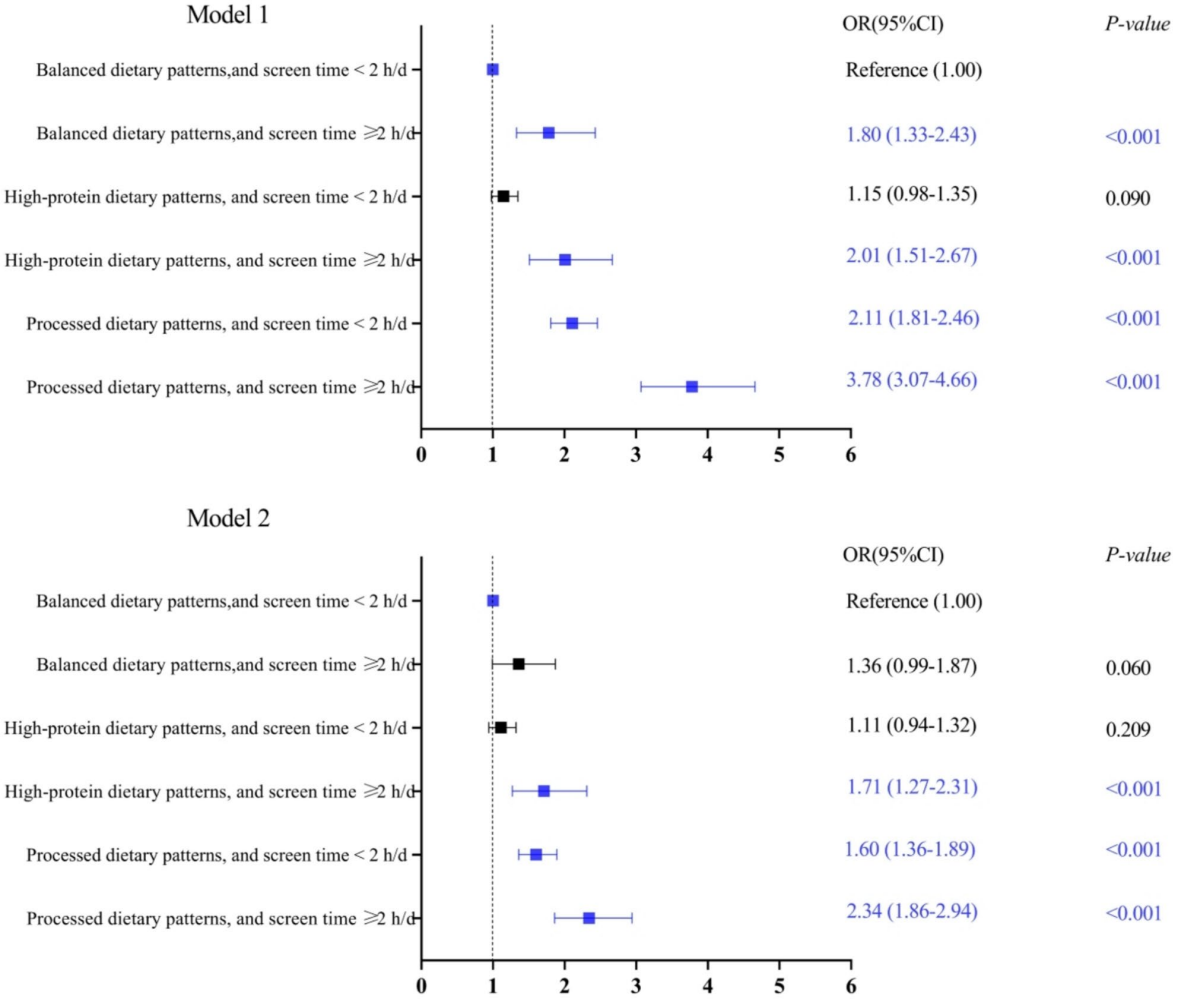
OR (95% CI) for the associations between co-exposure to dietary patterns and screen time with depressive symptoms in high school students. N, number; OR, odds ratio; CI, confidence interval. Model 1 has no adjustment variable. Model 2 is adjusted for sex, household registration type, residential location, only-child status, household economic status, paternal education level, maternal education level, household computer and internet access status, parental academic expectation, number of close friends, smoking status, drinking status, physical activity status

Compared to adolescents with balanced dietary patterns and screen time < 2 h/d, adolescents with high-protein dietary patterns and screen time ≥ 2 h/d (OR = 1.71, 95% CI 1.27–2.31), those with processed dietary patterns and screen time < 2 h/d (OR = 1.60, 95% CI 1.36–1.89), and those with processed dietary patterns and screen time ≥ 2 h/d (OR = 2.34, 95% CI 1.86–2.94) had 1.71 times, 1.60 times, and 2.34 times the risk of depressive symptoms, respectively (Fig. 1 and Table S3).

## Discussion

Our results indicate that adolescents exposed to both high-protein dietary patterns and screen time ≥ 2 h/d face a significantly elevated risk of depressive symptoms. Processed dietary patterns show a stronger correlation, increasing depressive symptom risks regardless of whether screen time falls below or exceeds 2 h/d. Firstly, the detection rate of depressive symptoms among middle school students in Shandong Province is 18.9%, closely aligned with prior findings by Hai (19.2%) [51] but significantly higher than the 13.6% prevalence reported in a large-scale survey of 10,770 Chinese adolescents [52], which reminds us that we need to pay high attention to the mental health problems of adolescents. Such differences in the detection rate of depressive symptoms may be related to factors such as the choice of measurement tools, classification criteria for depressive symptoms, and differences in the sample population.

This study through principal component analysis identified three main dietary patterns (balanced dietary patterns, high-protein dietary patterns, and processed dietary patterns). The results showed that among the participants, adopting processed dietary patterns was associated with an increased risk of depressive symptoms, and screen time ≥ 2 h/d was also significantly associated with an increased risk of depressive symptoms. Further analysis revealed that although no significant association between high-protein dietary patterns and the risk of depressive symptoms was observed in the Database of Youth Health, when high-protein dietary patterns coexists with screen time ≥ 2 h/d, it is significantly associated with an increased risk of depressive symptoms. This study further confirmed the significant association between co-exposure to dietary patterns and screen time with the risk of depressive symptoms.

In our study, we found that processed dietary patterns (sugar-sweetened beverages, western fast food, sweetened salt and sugar snacks and desserts, fried foods, instant noodles, processed meats, and seafood) were linked to an elevated risk of depressive symptoms, which was in line with previous studies [53, 54]. Processed dietary patterns may increase the risk of depressive symptoms through multiple mechanisms. This pattern is rich in oil, fat and sugar, and is usually accompanied by elevated levels of inflammatory factors [55]. Previous studies indicate that neuroimmune dysregulation and the release of inflammatory factors activate the peripheral immune system, which can subsequently induce endocrine dysfunction and contribute to the onset of depression [56]. In addition, with the continuous improvement of living standards, many teenagers are facing the problems of excessive consumption of desserts and insufficient intake of fresh fruits and vegetables [57]. Furthermore, our study also found that sugary beverages have the highest factor loading in processed dietary patterns. One potential biological explanation is that excessive consumption of sugar-sweetened beverages may contribute to depression via mechanisms involving mitochondrial damage, oxidative stress, and inflammatory responses [58, 59].

Our study observed no association of high-protein dietary patterns (dairy, common meat, fruits, processed meat, green vegetables, other vegetables, soybean products, and seafood) to adolescence with depressive symptoms in adolescence. Notably, the association between high-protein dietary patterns and depressive symptoms in adolescents remains complex and incompletely understood. While low-protein diets are beneficial for enhancing cognitive performance, excessive protein consumption has been associated with depression [60]. This is exemplified by milk, which provides the body with abundant amino acids and high-quality protein [61] and is widely recognized as an important protein source. Evidence suggests that individuals who consumed skimmed milk exhibited a lower risk of developing depressive symptoms compared with non-consumers, while whole milk consumption was associated with an elevated risk, particularly at intake levels between 129.63 g/d and 289.75 g/d [62]. A proposed biological mechanism for this association may involve insulin-like growth factor-1 (IGF-1), as milk consumption elevates circulating IGF-1 levels, and this factor has itself been positively correlated with depression [63]. Our study did not identify an association between high-protein dietary patterns and depressive symptoms, and the underlying reasons require further investigation.

This study revealed that screen time ≥ 2 h/d was an independent risk factor for depression in high school students. This aligns with prior evidence showing that spending more than 2 h on social media was associated with higher odds of feeling depressed often compared with spending 2 h or less [64]. Separately, Forte’s study found that social media use had the strongest association with depressive symptoms in the total sample and among females, whereas greater computer use was most strongly associated with depressive symptoms among males [25]. Epidemiological research results show that spending a large amount of time looking at screens increases the risk of symptoms, which was confirmed in a meta-analysis of a study that examined the association between screen time and risk of depression, with 1 h per day having the lowest risk and with the risk of depression increasing as the number of hours increases beyond 2 h. Excessive screen time may contribute to adolescent depression through some interrelated mechanisms. First, an upsurge in screen-based activities may displace time adolescents devote to interpersonal relationships, thus diminishing social interaction and potentially exacerbating depressive symptoms [65]. Second, extensive screen usage can impact teenage sleep patterns, thereby affecting academic performance and general wellbeing. Previous studies have shown that prolonged screen exposure, particularly at night, disrupts melatonin production due to blue light exposure, compromising sleep quality [66].

Our study suggests that dietary patterns and screen time are associated with depressive symptoms in high school students. Co-exposure analysis revealed that high-protein dietary patterns with screen time ≥ 2 h/d and processed dietary patterns with screen time ≥ 2 h/d were associated with an increased risk of depressive symptoms in high school students. During adolescence, hormone levels change dramatically, and individuals are highly susceptible to mood disorders and high-risk behaviors [67]. An unhealthy diet and excessive daily screen time are significant risk factors for obesity, diabetes, and other chronic diseases [68, 69], which in turn increase the risk of depressive symptoms [15, 70]. The coexistence of these two risk factors may trigger more serious adverse biological reactions, resulting in co-exposure effects greater than single exposure effects. These findings suggest that a healthy diet and reasonable screen time as controllable factors may be key factors for the comprehensive prevention and treatment of adolescent depressive symptoms.

The detection rate of depressive symptoms was higher among females compared to males, echoing the findings of previous studies [71] and potentially linked to estrogen level fluctuations during female puberty [72, 73]. Students from households with moderate economic status exhibited a higher depressive symptom detection rate than those from wealthier backgrounds. This difference might stem from greater parental attention and care in affluent families, leading to a lower incidence of depressive symptoms [74]. The elevated detection rate among non-only children could be attributed to factors such as unequal emotional support and care from parents, as well as the increased susceptibility of children to mental health issues in larger families [75]. Children of parents with higher education levels showed a lower detection rate of depressive symptoms, possibly reflecting more scientifically informed parenting approaches and a tendency towards rational cognition and emotional regulation [76], positively influencing children’s mental health. Our study also indicated that maintaining an adequate level of physical activity is associated with a reduced risk of depressive symptoms. Furthermore, smoking and drinking, identified as risk factors for depressive symptoms in numerous studies [77–79], were similarly found to be associated with increased depressive symptoms in our study.

Our research has several significant advantages. First, we used PCA methods to derive dietary patterns, investigating the associations of dietary patterns and screen time single and co-exposure with depressive symptoms. Second, this study has a relatively large sample size, ensuring good sample representatives. Third, the study incorporated relevant confounding factors into the regression model analysis, making the research conclusions more reliable.

This study has several limitations that need to be acknowledged. First, this study is a cross-sectional study and cannot confirm the causal associations between dietary patterns and screen time with depressive symptoms. Second, the frequency of food intake and screen time in this survey were both self-reported by the study subjects, which may be susceptible to recall bias. Third, the screen time survey encompasses only weekdays, from Monday to Friday, omitting weekend screen time, which could impact the accuracy and reliability of the data. Fourth, body mass index (BMI) is a significant confounding factor for depressive symptoms in adolescents [80]. Insufficient BMI data in this study, however, precluded its inclusion in the model.

## Conclusions

This study offers exploratory insights into the potential associations of dietary patterns and screen time, both individually and in combination, with depressive symptoms in adolescents. Our results suggest that adolescents who consume a high-protein diet and engage in screen time ≥ 2 h/d are at a significantly increased risk of experiencing depressive symptoms. Additionally, the impact of processed dietary patterns is particularly pronounced, as its association with both screen time < 2 h/d and screen time ≥ 2 h/d are linked to a heightened risk of depressive symptoms. Longitudinal studies are needed to better elucidate the causal relationships between dietary patterns, screen time, and depression.

## Supplementary Information

Supplementary Material 1

## Data Availability

Repository name: Database for Youth HealthDirect URL: https://www.ncmi.cn//phda/dataDetails.do?id=CSTR:17970.11.A0031.202107.209.V1.0License: CC BY 4.0, authorization from data owner needed.
