# Supplementary material for: Associations of dietary patterns and screen time with depressive symptoms among adolescents in Shandong Province, China
Source: BMC Public Health. 2025 Dec 20;26:317. doi: 10.1186/s12889-025-25976-z (PMC12838105; doi:10.1186/s12889-025-25976-z)
Supplement: Supplementary file 1 — Supplementary Material 1 [file 12889_2025_25976_MOESM1_ESM.docx]

**Supplementary Materials**

**Associations of dietary patterns and screen time with depressive symptoms among** **adolescents in Shandong Province, China**

Xiaomei Jiang^1, #^, Zhongyou Li ^1, #^, Pingjing Wen^1^, Yiyi Ling^1^, Hai Li ^1, *^, Jiongli Huang ^1, **^

^1^ *Department of Preventive Medicine, School of Public Health and Management, Guangxi University of Chinese Medicine, Nanning, 530200, China*

**Tables**: 3

**Figures**: 3

**Pages**: 8

**Tables**

**Table S1** The foods included in factor analysis.

**Table S2** Multiple factors analysis of depressive symptoms of the study subjects.

**Table S3** Associations between co-exposure to dietary patterns and screen time with depressive symptoms.

**Figures**

**Fig. S1** Flowchart of population in the study.

**Fig. S2** Directed acyclic graph of the relationship between dietary patterns, screen time and depressive symptoms.

**Fig. S3** Factor analysis scree plots.

Table S1 The foods included in factor analysis.

| ID | Foods |
| --- | --- |
| 1 | Sugar-sweetened beverages |
| 2 | Western fast food |
| 3 | Sweetened salt and sugar snacks and desserts |
| 4 | Fried food |
| 5 | Instant noodles |
| 6 | Processed meat |
| 7 | Red-orange vegetable |
| 8 | Tubers |
| 9 | Potatoes |
| 10 | Other vegetables |
| 11 | Green vegetables |
| 12 | Soybean products |
| 13 | Seafood |
| 14 | Dairy |
| 15 | Eggs |
| 16 | Common meat |
| 17 | Fruits |

Table S2 Multiple factors analysis of depressive symptoms of the study subjects.

| Groups | 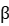 | S.E. | Wald 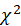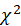 | *P-*value | OR | 95%CI | |
| --- | --- | --- | --- | --- | --- | --- | --- |
|  |  |  |  |  |  | Lower | Upper |
| **Sex** |  |  |  |  |  |  |  |
| male |  |  |  |  | 1.000 |  |  |
| Female | 0.465 | 0.064 | 52.500 | ＜0.001 | 1.592 | 1.404 | 1.806 |
| **Household registration type** |  |  |  |  |  |  |  |
| Non-agricultural |  |  |  |  | 1.000 |  |  |
| Agricultural | 0.004 | 0.074 | 0.003 | 0.954 | 1.004 | 0.869 | 1.160 |
| **Residential location** |  |  |  |  |  |  |  |
| Rural areas |  |  |  |  | 1.000 |  |  |
| Towns and townships | 0.016 | 0.088 | 0.035 | 0.852 | 1.017 | 0.856 | 1.208 |
| Urban-rural fringes | 0.237 | 0.123 | 3.717 | 0.054 | 1.268 | 0.996 | 1.614 |
| City and county urban areas | 0.100 | 0.085 | 1.395 | 0.238 | 1.105 | 0.936 | 1.304 |
| **Household economic status** |  |  |  |  |  |  |  |
| High |  |  |  |  | 1.000 |  |  |
| Moderate | -0.317 | 0.115 | 7.623 | 0.006 | 0.729 | 0.582 | 0.912 |
| Low | 0.207 | 0.130 | 2.534 | 0.111 | 1.230 | 0.953 | 1.587 |
| **Only-child status** |  |  |  |  |  |  |  |
| No |  |  |  |  | 1.000 |  |  |
| Yes | -0.177 | 0.075 | 5.558 | 0.018 | 0.838 | 0.724 | 0.971 |
| **Paternal education level** |  |  |  |  |  |  |  |
| Primary and below |  |  |  |  | 1.000 |  |  |
| Junior high school | -0.190 | 0.098 | 3.785 | 0.052 | 0.827 | 0.682 | 1.001 |
| High school and technical secondary school | -0.140 | 0.109 | 1.637 | 0.201 | 0.869 | 0.702 | 1.077 |
| Junior college or above | -0.197 | 0.139 | 2.012 | 0.156 | 0.821 | 0.625 | 1.078 |
| **Maternal education level** |  |  |  |  |  |  |  |
| Primary and below |  |  |  |  | 1.000 |  |  |
| Junior high school | -0.101 | 0.081 | 1.572 | 0.210 | 0.904 | 0.772 | 1.059 |
| High school and technical secondary school | -0.294 | 0.101 | 8.529 | 0.003 | 0.745 | 0.612 | 0.908 |
| Junior college or above | -0.371 | 0.140 | 7.062 | 0.008 | 0.690 | 0.525 | 0.907 |
| **Household computer and internet access status** |  |  |  |  |  |  |  |
| No computer or internet |  |  |  |  | 1.000 |  |  |
| Computer only | -0.063 | 0.175 | 0.130 | 0.719 | 0.939 | 0.666 | 1.323 |
| Both computer and internet | -0.093 | 0.109 | 0.730 | 0.393 | 0.911 | 0.735 | 1.129 |
| **Parental academic expectation** |  |  |  |  |  |  |  |
| No explicit expectation |  |  |  |  | 1.000 |  |  |
| Average level | -0.136 | 0.106 | 1.642 | 0.200 | 0.873 | 0.709 | 1.075 |
| Above average level | -0.227 | 0.079 | 8.235 | 0.004 | 0.797 | 0.683 | 0.931 |
| Top 5 in the class | -0.076 | 0.094 | 0.655 | 0.418 | 0.927 | 0.770 | 1.115 |
| **Number of close friends** |  |  |  |  |  |  |  |
| 0~1 |  |  |  |  | 1.000 |  |  |
| 2~4 | -0.156 | 0.069 | 5.052 | 0.025 | 0.856 | 0.747 | 0.980 |
| ≥5 | -0.148 | 0.080 | 3.380 | 0.066 | 0.863 | 0.737 | 1.010 |
| **Smoking status** |  |  |  |  |  |  |  |
| No |  |  |  |  | 1.000 |  |  |
| Yes | 0.586 | 0.086 | 46.766 | ＜0.001 | 1.797 | 1.519 | 2.126 |
| **Drinking status** |  |  |  |  |  |  |  |
| No |  |  |  |  | 1.000 |  |  |
| Yes | 0.915 | 0.070 | 170.612 | ＜0.001 | 2.496 | 2.176 | 2.863 |
| **Physical activity status (times/week)** |  |  |  |  |  |  |  |
| <1 |  |  |  |  | 1.000 |  |  |
| 1~2 | -0.636 | 0.070 | 82.895 | ＜0.001 | 0.529 | 0.462 | 0.607 |
| 3~4 | -0.906 | 0.096 | 88.168 | ＜0.001 | 0.404 | 0.335 | 0.488 |
| ≥5 | -0.745 | 0.106 | 49.796 | ＜0.001 | 0.475 | 0.386 | 0.584 |
| **Dietary patterns** |  |  |  |  |  |  |  |
| Balanced type |  |  |  |  | 1.000 |  |  |
| High-protein type | 0.126 | 0.079 | 2.530 | 0.112 | 1.134 | 0.971 | 1.325 |
| Processed type | 0.482 | 0.076 | 40.368 | ＜0.001 | 1.619 | 1.395 | 1.878 |
| **Screen time (h/d)** |  |  |  |  |  |  |  |
| <2 |  |  |  |  | 1.000 |  |  |
| ≥2 | 0.377 | 0.079 | 22.723 | ＜0.001 | 1.458 | 1.248 | 1.702 |
| Constant term | -1.032 | 0.212 | 23.726 | ＜0.001 | 0.356 | $-$ | $-$ |

Table S3 Associations between co-exposure to dietary patterns and screen time with depressive symptoms.

| Groups | 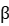 | S.E. | Wald 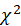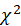 | *P-*value | OR | 95%CI | |
| --- | --- | --- | --- | --- | --- | --- | --- |
|  |  |  |  |  |  | Lower | Upper |
| **Sex** |  |  |  |  |  |  |  |
| Male |  |  |  |  | 1.000 |  |  |
| Female | 0.465 | 0.064 | 52.311 | ＜0.001 | 1.591 | 1.403 | 1.805 |
| **Household registration type** |  |  |  |  |  |  |  |
| Non-agricultural |  |  |  |  | 1.000 |  |  |
| Agricultural | 0.004 | 0.074 | 0.003 | 0.954 | 1.004 | 0.869 | 1.160 |
| **Residential location** |  |  |  |  |  |  |  |
| Rural areas |  |  |  |  | 1.000 |  |  |
| Towns and townships | 0.017 | 0.088 | 0.035 | 0.851 | 1.017 | 0.856 | 1.208 |
| Urban-rural fringes | 0.238 | 0.123 | 3.729 | 0.053 | 1.269 | 0.996 | 1.615 |
| City and county urban areas | 0.100 | 0.085 | 1.399 | 0.237 | 1.105 | 0.936 | 1.305 |
| **Household economic status** |  |  |  |  |  |  |  |
| High |  |  |  |  | 1.000 |  |  |
| Moderate | -0.315 | 0.115 | 7.552 | 0.006 | 0.729 | 0.582 | 0.913 |
| Low | 0.208 | 0.130 | 2.559 | 0.110 | 1.231 | 0.954 | 1.589 |
| **Only-child status** |  |  |  |  |  |  |  |
| No |  |  |  |  | 1.000 |  |  |
| Yes | -0.177 | 0.075 | 5.591 | 0.018 | 0.838 | 0.723 | 0.970 |
| **Paternal education level** |  |  |  |  |  |  |  |
| Primary and below |  |  |  |  | 1.000 |  |  |
| Junior high school | -0.190 | 0.098 | 3.775 | 0.052 | 0.827 | 0.682 | 1.002 |
| High school and technical secondary school | -0.140 | 0.109 | 1.634 | 0.201 | 0.870 | 0.702 | 1.077 |
| Junior college or above | -0.196 | 0.139 | 1.990 | 0.158 | 0.822 | 0.626 | 1.079 |
| **Maternal education level** |  |  |  |  |  |  |  |
| Primary and below |  |  |  |  | 1.000 |  |  |
| Junior high school | -0.101 | 0.081 | 1.564 | 0.211 | 0.904 | 0.772 | 1.059 |
| High school and technical secondary school | -0.295 | 0.101 | 8.586 | 0.003 | 0.745 | 0.611 | 0.907 |
| Junior college or above | -0.371 | 0.140 | 7.052 | 0.008 | 0.690 | 0.525 | 0.907 |
| **Household computer and internet access status** |  |  |  |  |  |  |  |
| No computer or internet |  |  |  |  | 1.000 |  |  |
| Computer only | -0.062 | 0.175 | 0.126 | 0.723 | 0.940 | 0.667 | 1.325 |
| Both computer and internet | -0.093 | 0.109 | 0.723 | 0.395 | 0.911 | 0.735 | 1.129 |
| **Parental academic expectation** |  |  |  |  |  |  |  |
| No explicit expectation |  |  |  |  | 1.000 |  |  |
| Average level | -0.135 | 0.106 | 1.621 | 0.203 | 0.874 | 0.710 | 1.076 |
| Above average level | -0.227 | 0.079 | 8.222 | 0.004 | 0.797 | 0.683 | 0.931 |
| Top 5 in the class | -0.075 | 0.094 | 0.640 | 0.424 | 0.927 | 0.771 | 1.116 |
| **Number of close friends** |  |  |  |  |  |  |  |
| 0~1 |  |  |  |  | 1.000 |  |  |
| 2~4 | -0.156 | 0.069 | 5.071 | 0.024 | 0.855 | 0.747 | 0.980 |
| ≥5 | -0.148 | 0.080 | 3.408 | 0.065 | 0.862 | 0.737 | 1.009 |
| **Smoking status** |  |  |  |  |  |  |  |
| No |  |  |  |  | 1.000 |  |  |
| Yes | 0.587 | 0.086 | 46.809 | ＜0.001 | 1.799 | 1.520 | 2.128 |
| **Drinking status** |  |  |  |  |  |  |  |
| No |  |  |  |  | 1.000 |  |  |
| Yes | 0.916 | 0.070 | 170.882 | ＜0.001 | 2.499 | 2.178 | 2.867 |
| **Physical activity status (times/week)** |  |  |  |  |  |  |  |
| <1 |  |  |  |  | 1.000 |  |  |
| 1~2 | -0.637 | 0.070 | 82.979 | ＜0.001 | 0.529 | 0.461 | 0.607 |
| 3~4 | -0.906 | 0.097 | 88.215 | ＜0.001 | 0.404 | 0.334 | 0.488 |
| ≥5 | -0.746 | 0.106 | 49.894 | ＜0.001 | 0.474 | 0.386 | 0.584 |
| **Combined exposure** |  |  |  |  |  |  |  |
| Balanced dietary patterns with screen time <2h/d |  |  |  |  | 1.000 |  |  |
| Balanced dietary patterns with screen time ≥2h/d | 0.307 | 0.164 | 3.528 | 0.060 | 1.360 | 0.987 | 1.874 |
| High-protein dietary patterns with screen time <2h/d | 0.108 | 0.086 | 1.575 | 0.209 | 1.114 | 0.941 | 1.318 |
| High-protein dietary patterns with screen time ≥2h/d | 0.538 | 0.153 | 12.306 | ＜0.001 | 1.713 | 1.268 | 2.313 |
| Processed dietary patterns with screen time <2h/d | 0.470 | 0.084 | 31.536 | ＜0.001 | 1.600 | 1.358 | 1.885 |
| Processed dietary patterns with screen time ≥2h/d | 0.850 | 0.116 | 53.799 | ＜0.001 | 2.339 | 1.864 | 2.936 |
| Constant term | -1.023 | 0.213 | 23.164 | ＜0.001 | 0.359 | $-$ | $-$ |


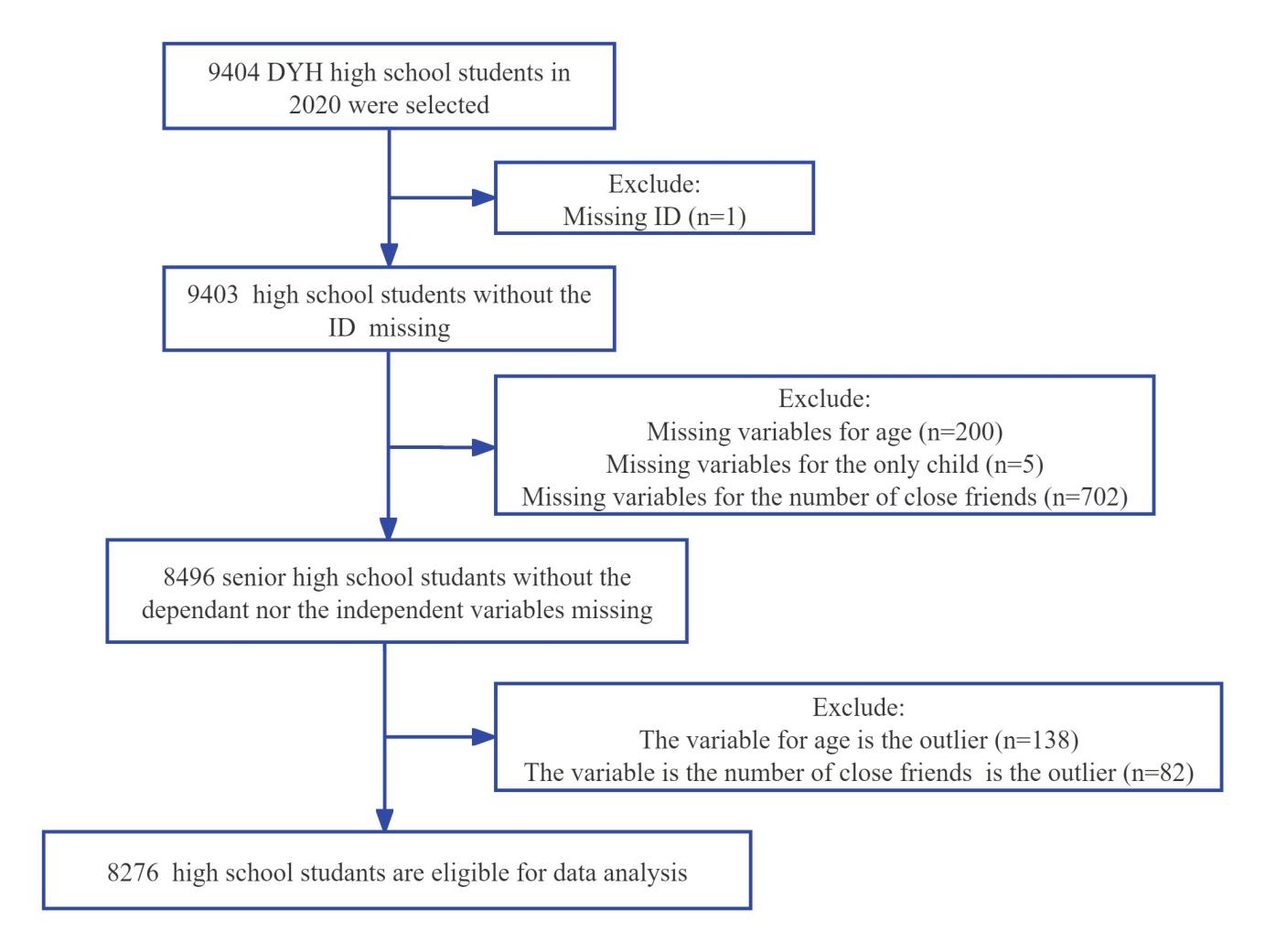


Fig. S1 Flowchart of population in the study


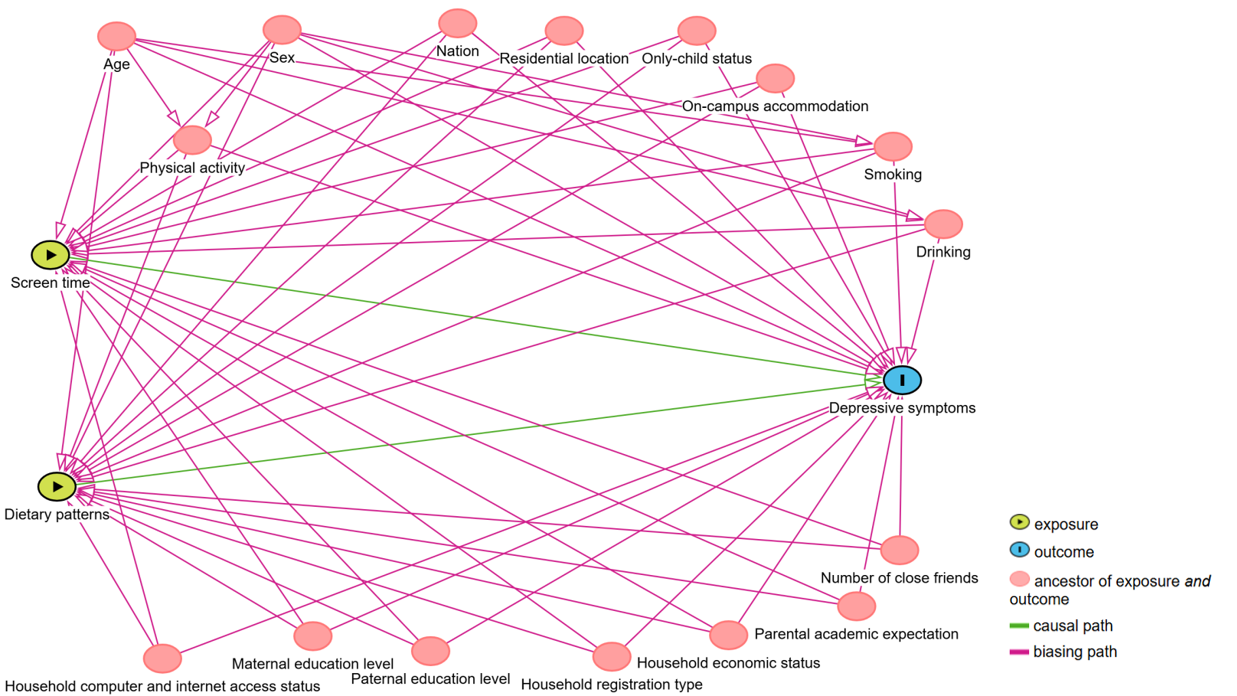


Fig. S2 Directed acyclic graph of the relationship between dietary patterns, screen time and depressive symptoms.


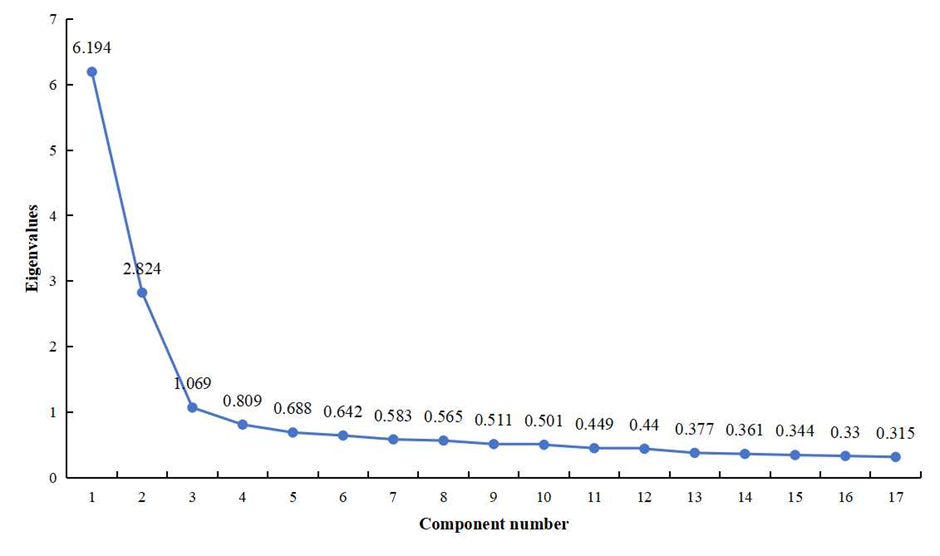


Fig. S3 Factor analysis scree plots.
